# Dolutegravir + Lamivudine vs. Dolutegravir + Tenofovir Disoproxil Fumarate/Emtricitabine: Very-Low-Level HIV-1 Replication through 144 Weeks in the GEMINI-1 and GEMINI-2 Studies

**DOI:** 10.3390/v16030405

**Published:** 2024-03-06

**Authors:** Mark Underwood, Rimgaile Urbaityte, Ruolan Wang, Joe Horton, James Oyee, Brian Wynne, Dainielle Fox, Bryn Jones, Choy Man, Jörg Sievers

**Affiliations:** 1 ViiV Healthcare, 406 Blackwell Street, Suite 300, Durham, NC 27701, USA; ruolan.h.wang@viivhealthcare.com (R.W.); dainielle.x.fox@viivhealthcare.com (D.F.);; 2 GSK, 980 Great West Road, Brentford, Middlesex TW8 9GS, UK; rimgaile.x.urbaityte@gsk.com (R.U.); james.x.oyee@gsk.com (J.O.); 3 Parexel, 2520 Meridian Parkway, Durham, NC 27713, USA; joseph.x.horton@gsk.com; 4 ViiV Healthcare, 980 Great West Road, Brentford, Middlesex TW8 9GS, UK; bryn.c.jones@viivhealthcare.com (B.J.);

**Keywords:** antiretroviral therapy, two-drug regimen, DTG + 3TC, HIV-1 infection, very-low-level viremia, virologic response

## Abstract

In GEMINI-1/-2, dolutegravir + lamivudine was non-inferior to dolutegravir + tenofovir disoproxil fumarate/emtricitabine (TDF/FTC) in achieving viral suppression (viral load [VL] < 50 copies/mL) in treatment-naive adults. Abbott’s RealTime HIV-1 assay provides quantitative VL (40–10,000,000 copies/mL) and qualitative target detected or target not detected (TND) for VL < 40 copies/mL. This post hoc analysis assessed very-low-level viremia and “blips” through Week 144. Proportions with VL < 40 copies/mL and TND are presented overall and by baseline VL and CD4+ cell count. “Blips” (single VL ≥ 50 to <200 copies/mL with adjacent values < 50 copies/mL) were assessed from Day 1 after VL suppression and from Weeks 48 through to 144. Proportions with TND increased through Week 48 and were similar between groups at all visits (Week 144: dolutegravir + lamivudine, 451/716 [63%]; dolutegravir + TDF/FTC, 465/717 [65%]). By observed analysis, TND rates were similar between groups across baseline subgroups. Through Week 144, proportions with ≥1 “blip” were generally comparable for dolutegravir + lamivudine vs. dolutegravir + TDF/FTC from Day 1 (15% vs. 20%) and from Week 48 (7% vs. 11%). Through 144 weeks, the proportions with TND or “blips” were similar between dolutegravir + lamivudine and the three-drug comparator, reinforcing the efficacy and durability of dolutegravir + lamivudine.

## 1. Introduction

Two-drug regimens (2DRs) as antiretroviral therapy (ART) for HIV-1 treatment can potentially reduce drug–drug interactions, long-term toxicities, and treatment costs compared with traditional three- or four-drug regimens [1,2]. Multiple 2DRs have demonstrated non-inferiority vs. three- or four-drug regimens in randomized controlled trials, with variable safety and tolerability profiles across time points ranging from 24 weeks to beyond 3 years depending on the antiretroviral agents included [2]. The 2DR dolutegravir/lamivudine (DTG/3TC) is a fixed-dose combination approved for HIV-1 treatment in ART-naive adults or as a switch option for virologically suppressed individuals [3]. Multiple HIV treatment guidelines recommend DTG/3TC as first-line ART for people with HIV-1 who have HIV-1 RNA < 500,000 copies/mL and no hepatitis B virus co-infection [4,5,6].

In the phase 3 GEMINI-1 and GEMINI-2 studies, the 2DR of DTG + 3TC taken as separate tablets demonstrated non-inferior efficacy to the three-drug regimen (3DR) DTG + tenofovir disoproxil fumarate/emtricitabine (TDF/FTC) in achieving virologic suppression (HIV-1 RNA < 50 copies/mL) in ART-naive adults at Week 48 [7]. The non-inferiority of DTG + 3TC vs. DTG + TDF/FTC was sustained through Week 144, demonstrating the durable efficacy of this 2DR [8]. Through 144 weeks, few participants in either treatment group met confirmed virologic withdrawal (CVW) criteria (DTG + 3TC, 2% [12/716]; DTG + TDF/FTC, 1% [9/717]), with no associated treatment-emergent resistance to integrase strand transfer inhibitors or nucleoside reverse transcriptase inhibitors detected [8]. One participant in the DTG + 3TC group who did not meet CVW criteria developed M184V at Week 132 and R263R/K at Week 144, conferring a 1.8-fold change in susceptibility to DTG, as previously described [8]. Virologic suppression rates at Week 144 were also generally comparable between DTG + 3TC and DTG + TDF/FTC when analyzed by baseline viral load (VL; >100,000 vs. ≤100,000 copies/mL) and CD4+ cell count (>200 vs. ≤200 cells/mm^3^).

Elevated VL events can occur after viral suppression has been achieved, such as virologic failure (confirmed VL ≥ 200 copies/mL), with persistent low-level viremia (consecutive VL between 50 and 200 copies/mL) and as VL “blips” (defined here as a single VL ≥ 50 and <200 copies/mL with adjacent VLs < 50 copies/mL) [4,9]. The Abbott RealTime HIV-1 assay reports quantitative VL results from 40 to 10,000,000 copies/mL, and VL results below the quantification limit use qualitative very-low-level HIV-1 replication [10] descriptors of target detected (TD) or, more stringently, target not detected (TND) [11]. In this post hoc analysis, we assessed very-low-level viremia by TD and TND as well as low-level quantitative viral replication, including “blips,” through 144 weeks in the GEMINI-1/-2 studies.

## 2. Materials and Methods

GEMINI-1 (NCT02831673) and GEMINI-2 (NCT02831764), which were identically designed, randomized, double-blind (through Week 96), and multicenter, phase 3 trials, were conducted at 187 centers in 21 countries. Study protocols were reviewed and approved by national, regional, or investigational center ethics committees or institutional review boards in accordance with the Declaration of Helsinki and the International Conference on Harmonization Guideline for Good Clinical Practice. All participants provided written informed consent before the initiation of the study procedures. Detailed methodology has been previously published [7].

Eligible participants were ART-naive (≤10 days of prior ART) adults with screening plasma HIV-1 RNA between 1000 and 500,000 copies/mL, no major reverse transcriptase or protease resistance-associated mutations, no hepatitis B virus co-infection, and no need for hepatitis C virus therapy. Participants were randomized 1:1 to receive DTG 50 mg + 3TC 300 mg once daily or DTG 50 mg + TDF 300 mg/FTC 200 mg once daily, stratified by screening VL (≤100,000 vs. >100,000 copies/mL) and screening CD4+ cell count (≤200 vs. >200 cells/mm^3^). Participants received study treatment in a double-blind randomized phase from Day 1 to Week 96 and in an open-label randomized phase from Weeks 96 to 148.

The primary endpoint in the GEMINI-1/-2 studies was the proportion of participants with plasma HIV-1 RNA < 50 copies/mL at Week 48 in the intention-to-treat–exposed (ITT-E) population using the US Food and Drug Administration Snapshot algorithm. Plasma HIV-1 RNA was assessed at baseline; Weeks 4, 8, 12, 16, 24, 36, and 48; and every 12 weeks thereafter through Week 144 using the Abbott RealTime HIV-1 assay (Abbott Molecular, Des Plaines, IL, USA). In this exploratory post hoc analysis, plasma HIV-1 RNA results were reported quantitatively for VLs from 40 to 10,000,000 copies/mL and qualitatively for VL < 40 copies/mL as TD (detected by the assay) or TND (not detected by the assay). The proportion of participants with VL < 40 copies/mL and TND status through Week 144 was analyzed based on the Snapshot algorithm using a Cochran–Mantel–Haenszel stratified analysis, adjusting for baseline VL (≤100,000 vs. >100,000 copies/mL), baseline CD4+ cell count (≤200 vs. >200 cells/mm^3^), and study (GEMINI-1 vs. GEMINI-2). Participant TND status at Week 144 was assessed in the overall ITT-E population and in the observed population (HIV-1 RNA < 50 copies/mL at last visit) overall and by baseline VL and CD4+ cell count. The ITT-E population included all randomized participants who received ≥1 dose of study treatment. The observed population included ITT-E participants with VL < 50 copies/mL at Week 144.

The time to TND status overall and by baseline VL subgroup was estimated using a non-parametric Kaplan–Meier method.

To assess elevated VL events after achieving suppression to <50 copies/mL, participants with VL ≥ 50 copies/mL were assigned as having “blips” if they had no VL ≥ 200 copies/mL and single VL from ≥50 to <200 copies/mL with adjacent values < 50 copies/mL. “Blips” were assessed from Day 1 to Week 144 after initial VL suppression to <50 copies/mL and from Weeks 48 through to 144 in the ITT-E population overall and by baseline VL and CD4+ cell count subgroups. “Blip” proportions were calculated based on the number of “blips” and available VL measurements within the analysis window. Multiple VL measurements from the same visit week were included, if applicable. Participants who never suppressed to <50 copies/mL or VL records from before and including the first suppression to <50 copies/mL were excluded.

## 3. Results

### 3.1. Participant Characteristics

In the pooled ITT-E population (DTG + 3TC, N = 716; DTG + TDF/FTC, N = 717), the baseline demographics were similar between treatment groups, as previously reported [7]. Most participants were men (85%) and White (68%), and the median (range) age was 33 (18–72) years. Similar proportions of participants in each group had baseline VL > 100,000 copies/mL (DTG + 3TC, 20% [140/716]; DTG + TDF/FTC, 21% [153/717]) and baseline CD4+ cell count ≤ 200 cells/mm^3^ (DTG + 3TC, 9% [63/716]; DTG + TDF/FTC, 8% [55/717]).

### 3.2. TND Assessments

**Figure 1:**
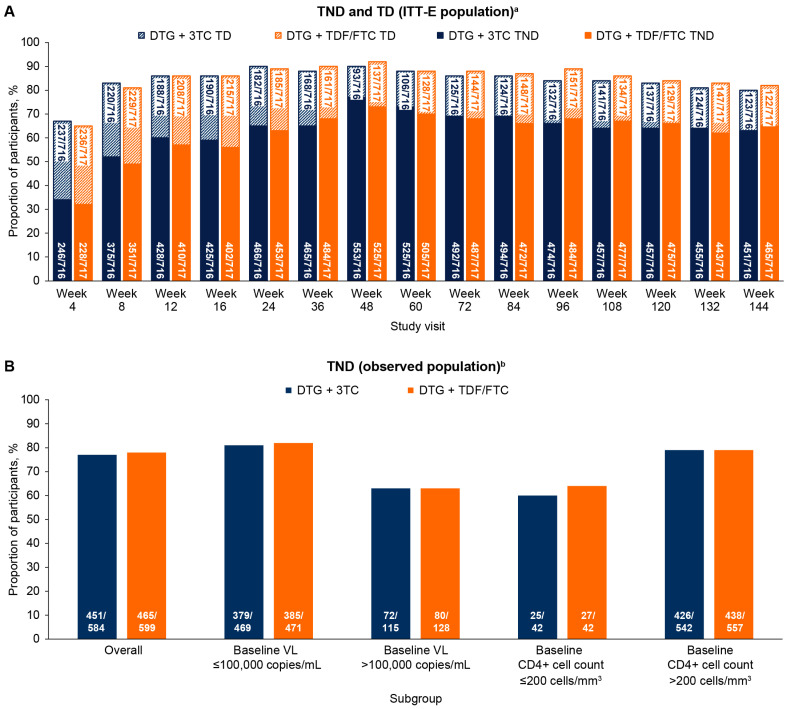
(**A**) Proportion of participants with TND and TD through Week 144 (ITT-E population). (**B**) Proportion of participants with TND by baseline subgroups at Week 144 (observed population). DTG, dolutegravir; FTC, emtricitabine; ITT-E, intention-to-treat–exposed; 3TC, lamivudine; TD, target detected; TDF, tenofovir disoproxil fumarate; TND, target not detected; VL, viral load. ^a^ The denominator at the base and top of each bar represents the total number of ITT-E participants. ^b^ The denominator at the base of each bar represents the total number of ITT-E participants with VL < 50 copies/mL at Week 144.

In the ITT-E population, the proportion of participants with TND gradually increased from Weeks 4 to 48 before stabilizing through Week 144, with similar proportions observed between treatment groups at all study visits (Figure 1A). At Week 144, 63% (451/716) of participants in the DTG + 3TC group and 65% (465/717) in the DTG + TDF/FTC group had TND. Converse to the TND findings, the proportion of participants with TD was highest in the earlier study visits, gradually decreased through Week 48, and stabilized thereafter in both treatment groups; 17% of participants in the DTG + 3TC (123/716) and DTG + TDF/FTC (122/717) groups had TD at Week 144.

In the observed population, at Week 144, the proportion of participants with TND was similar between the DTG + 3TC and DTG + TDF/FTC groups overall (77% [451/584] vs. 78% [465/599]) and when analyzed by baseline VL or CD4+ cell count subgroups (Figure 1B). Higher proportions with TND were observed in participants with baseline VL ≤ 100,000 copies/mL (DTG + 3TC, 81%; DTG + TDF/FTC, 82%) vs. >100,000 copies/mL (DTG + 3TC, 63%; DTG + TDF/FTC, 63%) and those with baseline CD4+ cell count > 200 cells/mm^3^ (DTG + 3TC, 79%; DTG + TDF/FTC, 79%) vs. <200 cells/mm^3^ (DTG + 3TC, 60%; DTG + TDF/FTC, 64%).

At Week 144, the median time to achieve TND was evaluated overall and by baseline VL in an observed analysis that included all participants with VL < 50 copies/mL at Week 144 (Figure S1). In the overall observed population, the median (95% CI) time to TND was 8 (not evaluable) weeks and 8 (~8, ~8) weeks for the DTG + 3TC and DTG + TDF/FTC groups, respectively. In participants with baseline VL ≤ 100,000 copies/mL, the median (95% CI) time to TND was 8 (~8, ~8) weeks for both treatment groups. Among participants with baseline VL > 100,000 copies/mL, the median (95% CI) time to TND was 16 (~16, ~24) weeks for the DTG + 3TC group and 24 (~24, ~35) weeks for the DTG + TDF/FTC group.

### 3.3. “Blip” Assessments

**Figure 2:**
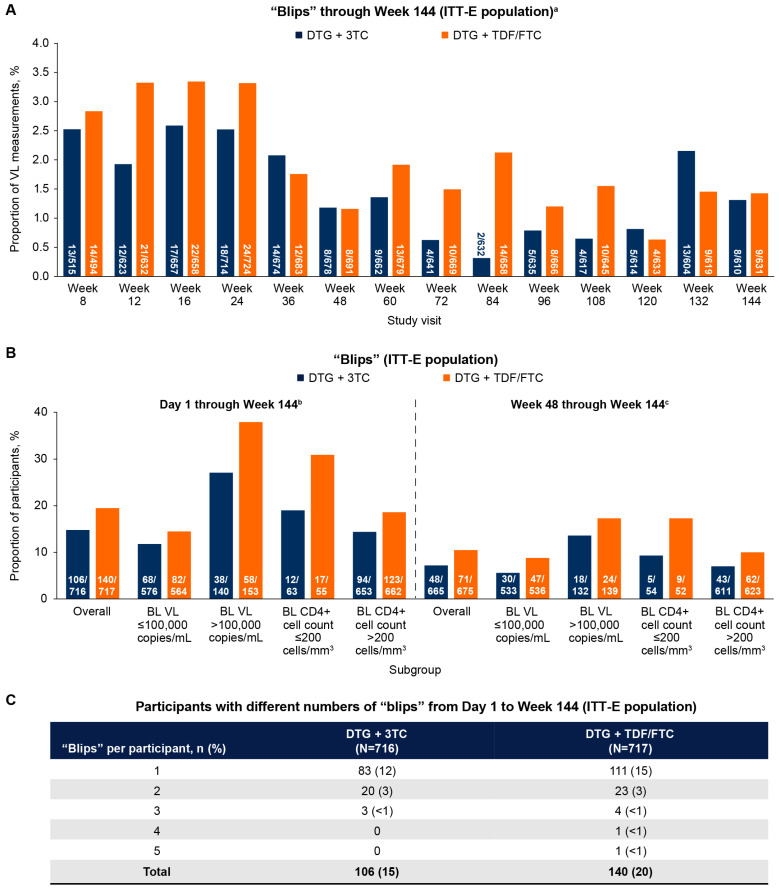
(**A**) “Blip” proportions through Week 144 (ITT-E population). (**B**) Proportion of participants with “blips” from Day 1 through Week 144 and from Week 48 through Week 144 overall and by baseline subgroups (ITT-E population). (**C**) Participants with different numbers of “blips” from Day 1 to Week 144 (ITT-E population). BL, baseline; DTG, dolutegravir; FTC, emtricitabine; ITT-E, intention-to-treat–exposed; 3TC, lamivudine; TDF, tenofovir disoproxil fumarate; VL, viral load. ^a^ The numerator in each bar represents the number of “blips” at each study visit. Participants could have >1 “blip”. The denominator at the base of each bar represents the total number of VL observations from all participants with data for the specified visit window. ^b^ The denominator at the base of each bar represents the total number of participants in the ITT-E population per category. ^c^ The denominator at the base of each bar represents the total participant population from Week 48 to Week 144.

In the ITT-E population, the proportions of available VL measurements with “blips” were higher in the earlier study visits and decreased around Weeks 36 to 48 (Figure 2A). “Blip” proportions were numerically lower with DTG + 3TC vs. DTG + TDF/FTC at most study visits, but were generally similar between groups through Week 144. At Week 144, the frequency of “blips” was 1% (8/610) in the DTG + 3TC group and 1% (9/631) in the DTG + TDF/FTC group.

The overall “blip” frequency was broadly lower when assessed from Weeks 48 to 144 (DTG + 3TC, 7%; DTG + TDF/FTC, 11%) than from Day 1 to Week 144 (DTG + 3TC, 15%; DTG + TDF/FTC, 20%; Figure 2B). When analyzed by baseline VL and CD4+ cell count, the proportions of participants with ≥1 “blip” were numerically lower with DTG + 3TC vs. DTG + TDF/FTC, with the greatest differences observed from Day 1 to Week 144 in participants with baseline VL > 100,000 copies/mL (27% vs. 38%) or CD4+ cell count ≤ 200 cells/mm^3^ (19% vs. 31%).

Overall, 106 (15%) participants in the DTG + 3TC group and 140 (20%) in the DTG + TDF/FTC group had “blips” from Day 1 to Week 144 (Figure 2C). Most participants had only one “blip,” and this proportion was lower in the DTG + 3TC (n = 83, 12%) vs. DTG + TDF/FTC group (n = 111, 15%). No “blips” were observed in participants meeting the CVW criteria in either treatment group.

## 4. Discussion

In this post hoc analysis of the GEMINI-1/-2 studies, similar proportions of participants treated with the 2DR DTG + 3TC had very-low-level VL and TND, overall and by baseline VL and CD4+ cell count subgroups, compared with those treated with the 3DR DTG + TDF/FTC through 144 weeks. In addition, the occurrence of VL “blips” was generally comparable between treatment groups, although numerically lower with DTG + 3TC. These data provide further support for the potency and durability of DTG + 3TC.

The implications of very-low-level qualitative HIV-1 replication (TD and TND) remain exploratory and without established or apparent clinical consequences. Very-low-level viremia can persist during virologic suppression with ART through replication-competent reservoirs of infected cells that decay at differing rates across various anatomic sites, making it difficult to ascertain the source(s) and mechanism(s) responsible [12]. The proportions of participants with VL < 40 copies/mL and TND increased over time through Week 48 in both treatment groups, reflective of the increasing virologic suppression rates observed through 48 weeks after initiating ART in this treatment-naive population [7]. Thereafter, proportions with TND generally stabilized and remained similar between treatment groups through Week 144, consistent with the long-term virologic suppression rates reported in the GEMINI-1/-2 studies [8]. In the overall observed population, the median time to achieve TND was similarly rapid with the 2DR DTG + 3TC and the 3DR DTG + TDF/FTC (8 weeks in both treatment groups). In an observed analysis by baseline VL and CD4+ cell count, the proportion of participants with TND was similar between treatment groups at Week 144. However, among participants with baseline VL > 100,000 copies/mL, the median time to TND was numerically shorter for DTG + 3TC vs. DTG + TDF/FTC (16 vs. 24 weeks), further supporting the efficacy of 2DRs, including among individuals with high VL at ART initiation.

Low-level single HIV-1 VL elevations, known as “blips,” are generally considered to be not clinically impactful and likely represent variation around a suppressed VL level [4,13,14]. The results from this analysis support the lack of clinical significance associated with “blips,” as no “blips” were observed among the small number of participants meeting the CVW criteria (DTG + 3TC, n = 12; DTG + TDF/FTC, n = 9) [8]. The proportion of participants with “blips” through 144 weeks was generally similar between treatment groups in the overall ITT-E population. Although higher “blip” frequencies were observed with DTG + TDF/FTC from Day 1 to Week 144 among participants with baseline VL > 100,000 copies/mL or CD4+ cell count ≤ 200 cells/mm^3^, none met the CVW criteria; thus, any potential relationship between “blips” and treatment failure in participants with these more advanced disease characteristics remains uncertain. In addition, it is possible that the numerically greater number of “blips” in the DTG + TDF/FTC group at Week 24 onward might reflect participants with a propensity to have “blips” being differentially withdrawn from the DTG + 3TC group compared with the DTG + TDF/FTC group.

This analysis has limitations. Most participants in the GEMINI-1/-2 studies were men, White, and aged < 50 years, which may limit the generalizability of these findings. Through 148 weeks in GEMINI-1/-2, 257 of 1433 (18%) participants in the ITT-E population discontinued [8], which may have contributed to lower TND rates at later study visits. As baseline VL subgroups were limited to ≤100,000 vs. >100,000 copies/mL, long-term data on very-low-level HIV-1 replication or VL “blips” were not differentially reported for individuals with higher baseline VLs up to 500,000 copies/mL. The number of participants with baseline CD4+ cell count ≤ 200 cells/mm^3^ was low, which limits the interpretation of results in individuals with advanced HIV disease.

This post hoc analysis demonstrated comparable virologic responses between the 2DR DTG + 3TC and the 3DR DTG + TDF/FTC through 144 weeks using the more stringent VL measure represented by TND. Isolated VLs ≥50 to <200 copies/mL (also known as “blips”) are of uncertain predictive value for subsequent treatment failure; however, the similar occurrence of “blips” between treatment groups suggests that the 2DR DTG + 3TC was as effective as the 3DR DTG + TDF/FTC in suppressing the viral reservoir in the GEMINI-1 and -2 studies. Overall, these data continue to demonstrate the efficacy, potency, and durability of DTG + 3TC in ART-naive adults.

## Data Availability

Anonymized individual participant data and study documents can be requested for further research from www.clinicalstudydatarequest.com.

## Supplementary Materials

The following supporting information can be downloaded at: https://www.mdpi.com/article/10.3390/v16030405/s1, Figure S1: Time to VL < 40 copies/mL and TND status (A) overall and (B,C) by baseline VL subgroups (observed analysis).
