# Supplementary material for: Dolutegravir + Lamivudine vs. Dolutegravir + Tenofovir Disoproxil Fumarate/Emtricitabine: Very-Low-Level HIV-1 Replication through 144 Weeks in the GEMINI-1 and GEMINI-2 Studies
Source: Viruses. 2024 Mar 6;16(3):405. doi: 10.3390/v16030405 (PMC10976086; doi:10.3390/v16030405)
Supplement: Supplementary file 1 [file viruses-16-00405-s001.zip › viruses-2884609-supplementary.pdf]

Figure S1. Time to VL <40 copies/mL and TND status **(A)** overall and **(B-C)** by baseline VL subgroups (observed analysis). All participants with VL <50 copies/mL at Week 144 were included. DTG, dolutegravir; FTC, emtricitabine; NE, not evaluable; 3TC, lamivudine; TDF, tenofovir disoproxil fumarate; TND, target not detected; VL, viral load.

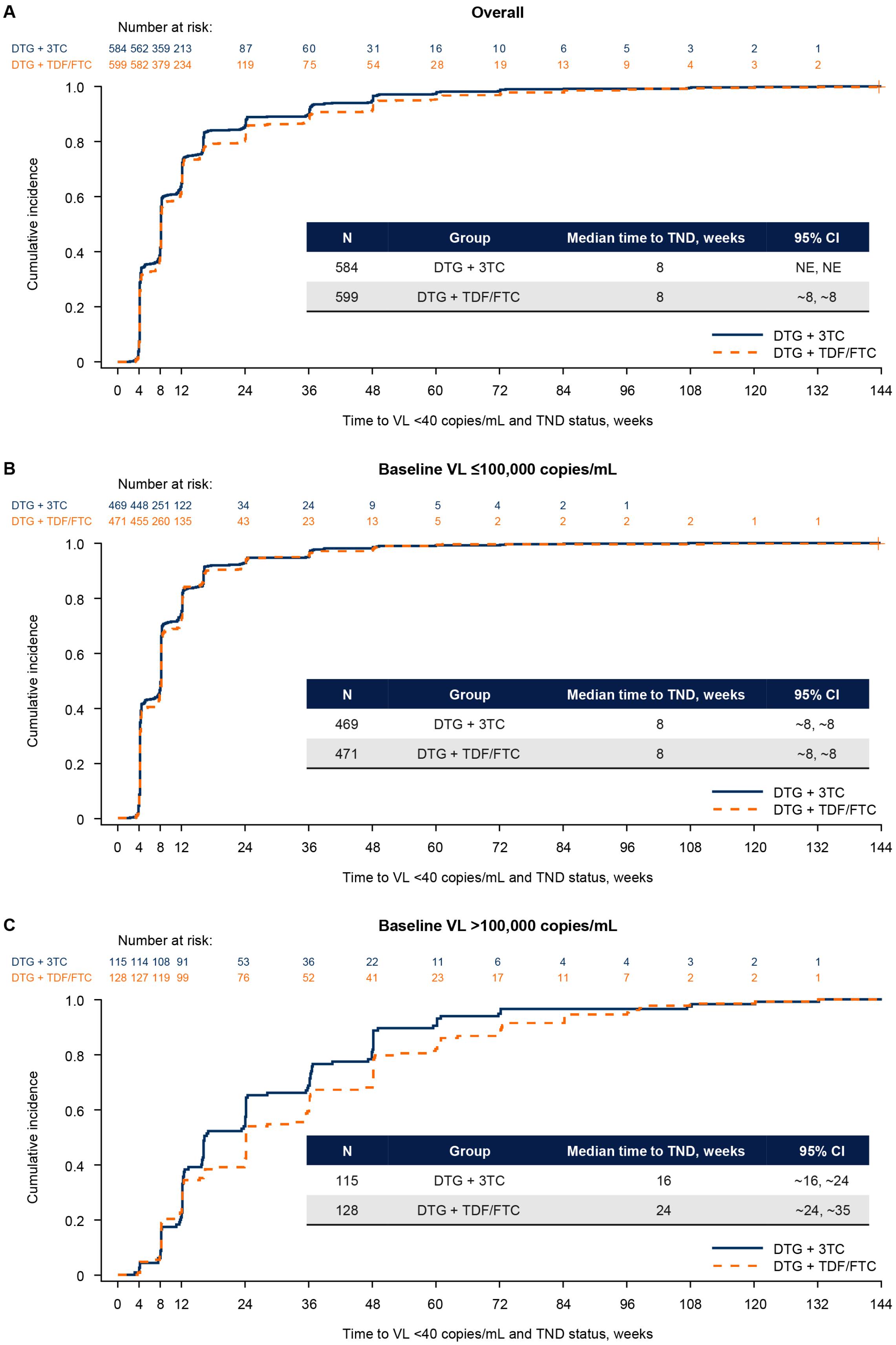
A
Overall
Number at risk:
DTG + 3TC 584 562 359 213 87 60 31 16 10 6 5 3 2 1
DTG + TDF/FTC 599 582 379 234 119 75 54 28 19 13 9 4 3 2
Cumulative incidence
1.0
0.8
0.6
0.4
0.2
0
N | Group | Median time to TND, weeks | 95% CI
584 | DTG + 3TC | 8 | NE, NE
599 | DTG + TDF/FTC | 8 | ~8, ~8
DTG + 3TC
DTG + TDF/FTC
0 4 8 12 24 36 48 60 72 84 96 108 120 132 144
Time to VL <40 copies/mL and TND status, weeks
B
Baseline VL ≤100,000 copies/mL
Number at risk:
DTG + 3TC 469 448 251 122 34 24 9 5 4 2 1
DTG + TDF/FTC 471 455 260 135 43 23 13 5 2 2 2 2 1 1
Cumulative incidence
1.0
0.8
0.6
0.4
0.2
0
N | Group | Median time to TND, weeks | 95% CI
469 | DTG + 3TC | 8 | ~8, ~8
471 | DTG + TDF/FTC | 8 | ~8, ~8
DTG + 3TC
DTG + TDF/FTC
0 4 8 12 24 36 48 60 72 84 96 108 120 132 144
Time to VL <40 copies/mL and TND status, weeks
C
Baseline VL >100,000 copies/mL
Number at risk:
DTG + 3TC 115 114 108 91 53 36 22 11 6 4 4 3 2 1
DTG + TDF/FTC 128 127 119 99 76 52 41 23 17 11 7 2 2 1
Cumulative incidence
1.0
0.8
0.6
0.4
0.2
0
N | Group | Median time to TND, weeks | 95% CI
115 | DTG + 3TC | 16 | ~16, ~24
128 | DTG + TDF/FTC | 24 | ~24, ~35
DTG + 3TC
DTG + TDF/FTC
0 4 8 12 24 36 48 60 72 84 96 108 120 132 144
Time to VL <40 copies/mL and TND status, weeks
